# Resolution of Persistent Chylothorax With a Ketogenic Diet: A Case Report

**DOI:** 10.7759/cureus.64144

**Published:** 2024-07-09

**Authors:** Jack C Redick, Christy Kesslering

**Affiliations:** 1 Internal Medicine, The Ohio State University College of Medicine, Columbus, USA; 2 Metabolic Health, KessRX, Elmhurst, USA

**Keywords:** insulin resistance, ketogenic diet, chylomicrons, triglycerides, thoracic duct ligation, pleural effusion, chylothorax

## Abstract

This is the first case report of a very low-carbohydrate, high-fat ketogenic diet for the treatment of chylothorax. A 61-year-old female with recurrent chylothorax following thoracic surgery was refractory to a very low-fat diet managed by a hospital dietitian. She required repeated palliative thoracentesis to the point where she was scheduled for a thoracic duct embolization. Prior to the embolization, she was placed on a very low-carbohydrate (<20 total grams daily), high-fat, ketogenic diet. Metabolic markers and imaging were obtained regularly. The patient had improvements in her serum triglycerides, triglyceride/HDL ratio, and triglyceride-glucose index, as well as clinical and radiographic improvements in her chylothorax as assessed by a chest X-ray and CT scan. Within three months of starting her ketogenic diet, imaging revealed complete resolution of the chylous pleural effusion. This case suggests that metabolic optimization to decrease insulin resistance, improve chylomicron metabolism, decrease lymphatic permeability, and lower serum triglycerides, as occurs with a ketogenic diet, should be considered for conservative treatment of chylothorax and warrants further study.

## Introduction

Chylothorax is an accumulation of chyle in the pleural space. A chylothorax can be spontaneous, infectious, or malignant, but most commonly today, it is the result of a traumatic injury to the thoracic duct. These injuries most commonly occur as a complication of thoracic surgery [1,2]. In an adult, the thoracic duct transports up to four liters of chyle daily. Medication, diet, movement, and gut function may influence the flow of lymph through the thoracic duct [3].

Clinically, patients typically present with dyspnea, cough, and/or chest pain. These pleural effusions tend to be right-sided due to the anatomy of the thoracic duct. Chyle is typically a milky-appearing fluid composed of chylomicrons, long-chain triglycerides, cholesterol esters, phospholipids, immunoglobulins, and lymphocytes carried through the lymphatic system [1]. An effusion triglyceride level over 110 mg/dL is diagnostic of a chylothorax [4]. Persistent leaks can lead to malnutrition and immunologic compromise [1]. Although quite rare, it has also been shown that the chylous fluid may have much lower triglyceride levels in the setting of malnutrition and/or fasting [5].

The management of chylothorax is controversial. Some advocate for conservative measures like palliative thoracentesis, chest tube fasting, total parenteral nutrition with or without medium-chain triglycerides (MCTs), or low-fat diets with or without MCTs, while others advocate for early surgical intervention, such as surgical ligation or embolization of the thoracic duct. 

In an attempt to reduce the flow of lymph through the thoracic duct, MCTs have been the preferred source of fat for patients with chylothorax since they are absorbed directly into the portal system without going through the intestinal lymph vessels and thus the thoracic duct [6]. The data supporting a low-fat diet with or without MCTs has yielded mixed responses; some show benefit while others show little improvement in chylous effusions [7-10]. 

Traditionally, it has been thought that diets high in fat and cholesterol increase serum fatty acids and serum cholesterol, which then increase the lymphatic load in the thoracic duct, leading to an increased chylous pleural effusion. Therefore, the dietary approach has always been to restrict dietary fat and cholesterol.

Dietary fat has different effects on plasma triglycerides, depending on the type of diet used. It has been shown that serum triglycerides are significantly reduced in the setting of a carbohydrate-restricted diet [11]; thus, a low-carbohydrate diet may have a positive effect on chylous effusions.

To the best of our knowledge, a high-fat, low-carbohydrate ketogenic diet has not been reported for the conservative treatment of chylothorax, as a high-fat diet has been theorized to worsen the effusion. However, as this case demonstrates, a high-fat diet in the setting of a very low-carbohydrate diet may have the opposite effect of a high-fat diet otherwise.

This case was originally presented as a poster on January 26, 2024, at the Metabolic Health Summit in Clearwater, FL.

## Case presentation

A 61-year-old female with a prior history of endometriosis underwent a hysterectomy and bilateral salpingo-oophorectomy in 2012. Due to the extent of the disease, her left ovary was removed in pieces. She was seen regularly by her gynecologist, and on a routine pelvic exam in 2019, she was found to have a new left pelvic mass. She underwent CT imaging of the abdomen and pelvis, which revealed a mass in the remnant left adnexa. She again underwent surgery for recurrent endometriosis. Incidentally, the 2019 scan showed a 1.5 cm left lower lobe lung nodule, a 0.6 cm right apical lung nodule, and a 1.6 cm right middle lobe lung nodule. A PET scan was done, revealing borderline uptake in the right middle lobe nodule and no uptake in the right apical or left lower lobe nodules. A right middle lobe biopsy was attempted but was unsuccessful, per the surgeon’s notes, so she was followed by routine imaging.

In August 2022, in addition to further recurrence and progression of her endometriosis, her CT scan revealed increased growth in the right middle lobe lesion with no significant changes in the right apical or left lower lobe lesions. A PET revealed increased activity in the right middle lobe lesion, now measuring 2.3 cm, without uptake in the other two lesions. Again, a CT biopsy attempt was made and was unsuccessful, and thus, on November 28, 2022, she underwent right middle lobe wedge resection with nodal sampling (8 total nodes removed). Pathology revealed a 4.2 cm grade 3 meningioma with negative margins, negative nodes, and negative pleural fluid. As primary meningioma of the lung is exceedingly rare, she underwent additional workups to look for a primary site. MRI scans of the brain and spine were done, which were all negative.

**Figure 1:**
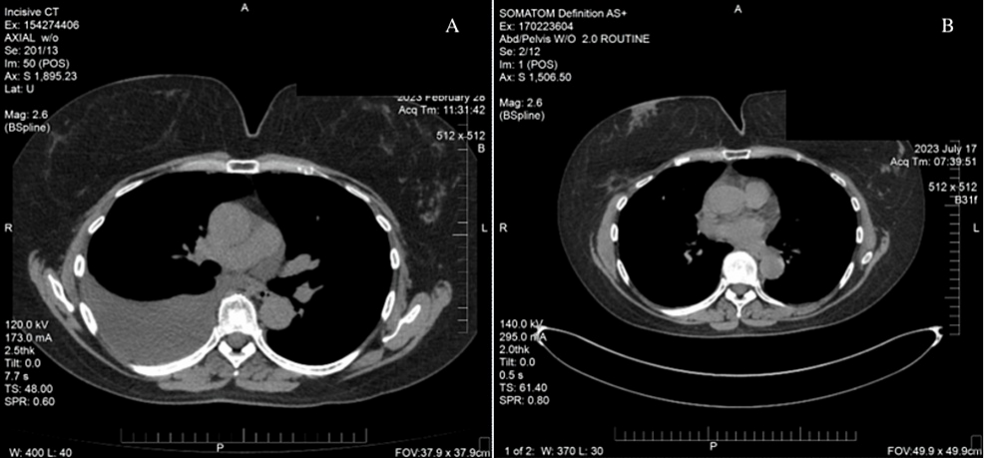
CT scan images showing right-sided pleural effusion resolution (A) At symptomatic presentation on February 28, 2023; (B) showing complete resolution on July 17, 2023.

On December 8, 2022, a routine post-operative chest X-ray revealed a moderate right pleural effusion. On December 19, 2022, she underwent thoracentesis and had 300 mL of cloudy, white fluid drained. No testing was done on the fluid at this time. In February 2023, she presented with significant and progressive shortness of breath and cough. A CT scan on February 28, 2023, revealed a large right-sided pleural effusion (as well as stable left lower lobe and right apical lung nodules) (Figure 1A). She underwent a second thoracentesis on March 3, 2023. A total of 1100 mL of cloudy, white fluid was drained. Cytology was negative for malignancy or other pathology. The fluid did reveal chronic inflammatory changes and was positive for triglycerides at 3,885 mg/dL. The fluid was not tested for cholesterol.

**Figure 2:**
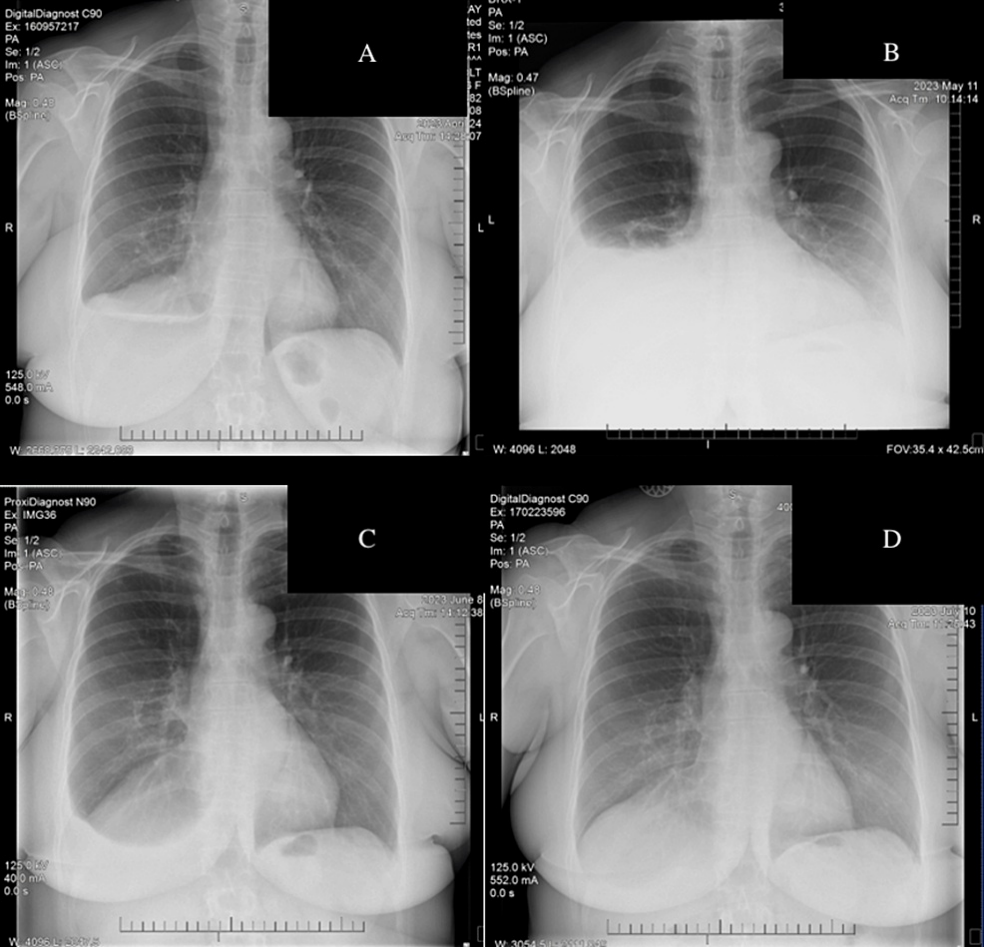
Chest X-rays showing right-sided pleural effusion resolution (A) After last thoracentesis, (B) four weeks after changing diet and two weeks after last thoracentesis, (C) seven weeks after changing diet and five weeks after last thoracentesis, (D) eleven weeks after changing diet and nine weeks after last thoracentesis.

The initial recommendation for her chylothorax was conservative management with dietary changes. The patient met with a dietitian and was instructed on how to follow a low-fat diet. The patient states that she followed these recommendations closely. Her diet consisted of lean fish, egg whites, whole grains, vegetables, fruit, low-fat yogurt, and protein powder smoothies. Unfortunately, her respiratory symptoms returned within two months, and she was again found to have a significant pleural effusion. She underwent her third thoracentesis on April 24, 2023, and had 1100 mL of cloudy, white fluid drained (Figure 2A). Given the continued leak, a follow-up chest X-ray was scheduled for two weeks (Figure 2B), and a thoracic duct embolization procedure was scheduled for late June.

On April 20, 2023, just prior to her April thoracentesis, the patient sought outside consultation regarding diet and lifestyle management to improve her metabolic health given her weight (207 lbs, BMI 31.5), diagnosis of endometriosis, and meningioma, all of which are associated with insulin resistance [12-14]. 

At the time of consultation, as well as at follow-up visits, labs were obtained to assess metabolic markers (Table 1). And although her fasting glucose, insulin, and Homeostatic Model Assessment of Insulin Resistance (HOMA-IR) were not elevated, her Triglyceride-Glucose Index (TyG index, calculated by Ln[glucose x triglycerides]/2) and her triglyceride/HDL ratio (TG/HDL ratio) were significantly elevated at baseline and consistent with insulin resistance [15,16].

**Table 1:** Insulin resistance markers showing gradual improvements to optimal levels over two and a half months on a ketogenic diet. HDL: high-density lipoprotein, HOMA-IR: Homeostatic Model Assessment of Insulin Resistance, TG/HDL ratio: triglyceride/HDL ratio, TyG Index: Triglyceride-Glucose Index.

	Reference values	April 7, 2023	May 12, 2023	June 27, 2023
Fasting glucose (mg/dL)	<85	89	87	89
Fasting insulin (μIU/mL)	<4	3.7	2.1	1.5
Triglycerides (mg/dL)	<70	118	97	62
HDL (mg/dL)	60-100	34	44	65
HOMA-IR	<1	0.8	0.5	0.3
TG/HDL ratio	<1.2	3.5	2.2	1
TyG Index	<4.49	4.63	4.52	4.31

A discussion was had at that time about switching her diet from her current low-fat diet to another diet often used in chylothorax using high medium-chain triglycerides (MCT), with lower glycemic, low-fat food choices until embolization, at which time she would move to a carbohydrate-restricted (<20 g daily), high-fat ketogenic diet versus moving to a carbohydrate-restricted, high-fat ketogenic diet immediately. The rationale for using a carbohydrate-restricted, high-fat ketogenic diet is discussed below. The patient opted to start a carbohydrate-restricted ketogenic diet on April 21, 2023. 

Upon returning to the radiology department on May 11, 2023, for her routine follow-up chest X-ray, she was found to have a moderate effusion, which was less than expected. As she remained asymptomatic, this was not drained, and a one-month thoracentesis was scheduled. On June 8, 2023, she presented for her scheduled thoracentesis, although she again remained asymptomatic. When she underwent ultrasound imaging at the procedure, no significant fluid was detected, and thus a repeat chest X-ray was done (Figure 2C). This confirmed significant regression of her pleural effusion, and thus her thoracentesis was cancelled, as was the scheduled embolization.

She returned one month later, on July 10, 2023, for another follow-up chest X-ray, which revealed a continued decrease in the size of the residual right-sided pleural effusion (Figure 2D). Further evaluation with a CT scan of the chest on July 17, 2023, also showed marked improvement in the effusion (Figure 1B).

The patient used a diet tracking app, Cronometer, to assess her adherence to the recommended ketogenic diet. She consistently hit a daily macronutrient breakdown of 100 g protein, 130 g fat, and 15 g total carbohydrates per day. Per the review of the patient's Cronometer, she consumed mostly dairy, animal protein sources, coconut oil, and some MCT oil with her morning coffee. Her adherence was monitored with a Keto-Mojo meter to track her glucose and ketones. Her ketones ranged from 0.5 to 1.5 mmol/L, with an average of 1.1 mmol/L, indicating she was in nutritional ketosis. 

A laboratory assessment of her metabolic markers was done prior to and after her switch to a ketogenic diet. We measured fasting values of glucose, insulin, triglycerides, and high-density lipoprotein (HDL) and used these values to calculate the Homeostatic Model Assessment of Insulin Resistance (HOMA-IR), triglyceride/HDL ratio, and Triglyceride-Glucose Index (TyG index), which have all been shown to be indicators of insulin resistance. Despite having an optimal HOMA-IR, both her TG/HDL ratio and TyG index were consistent with insulin resistance.

She had incremental improvements in all of her markers except for fasting glucose, which was within the normal range at baseline and remained stable (Table 1). Supplementation with vitamin D3, omega 3, and magnesium was also started with the diet changes. The patient noted a 6.9 kg weight loss related to the diet changes over the 2.5-month period.

## Discussion

The patient developed a chylous effusion following thoracic surgery, for which she was placed on a low-fat diet. Her prescribed low-fat diet, overseen by a registered dietician, was unable to manage her symptoms; thus, she underwent multiple palliative thoracentesis. Her effusion was refractory despite standard-of-care treatment, so her doctors planned a thoracic duct embolization to ameliorate her symptoms. At that time, the patient sought consultation and counseling on lifestyle modifications to address various conditions related to metabolic dysfunction, including endometriosis, obesity, and meningioma [12-14]. After shared decision-making, the patient decided to begin a very low-carbohydrate ketogenic diet. The patient was aware that this diet change was contrary to the advice of her medical team’s recommendation of a low-fat diet; however, she desired to improve her metabolic health, knowing that her embolization was already scheduled and the low-fat diet was not improving her chylothorax. Less than one month after beginning the very low-carb (less than 20 g of carbs per day) ketogenic diet, the patient noticed a complete resolution of her symptoms before her scheduled procedure could take place. Further, the resolution of the chylothorax, monitored via chest radiographs, appeared to correlate to the degree to which she was able to reverse her underlying insulin resistance (Figure 2 and Table 1).

Currently, the general philosophy in medicine is that dietary fat (especially saturated fat) is responsible for many chronic diseases (like obesity, diabetes, and heart disease); however, this idea continues to be challenged [11]. As opposed to the current standard American diet, which is both high in fat and high in carbohydrate, there are significant differences in metabolic processes when fat consumption is high in the setting of significant carbohydrate restriction. The treatment of chylothorax with diet has followed much the same recommendations as obesity. Eat low-fat to decrease fat in/on the body. This current standard of therapy would suggest that any form of high-fat diet would worsen chylothorax. As we have seen in this case, challenging that idea is warranted. Rather, analysis of the research on the matter suggests insulin resistance plays a much greater role than the amount of fat consumed.

Small and medium-chain triglycerides can be readily broken down in the small intestine into free fatty acids for transport and utilization in the portal circulation, whereas dietary large-chain triglycerides combine with apoB-48 and cholesterol esters to form chylomicrons, which are transported in the lymphatic system. Chyle, the fluid of the lymphatic system, is primarily formed by chylomicrons [1]. Thus, increased chylomicrons can increase lymphatic fluid. Recent studies have shown that increased chylomicron formation is not simply dependent on the amount of fat consumed. Metabolic function plays an important regulatory role [17]. Both fasting and postprandial triglycerides, as well as apoB-48 (the two primary building blocks of chylomicrons), are increased in obesity, dyslipidemia, and insulin resistance [17]. Insulin resistance was shown to increase chylomicron production and secretion in the intestine [17]. It was found that in postmenopausal women (our patient’s demographic), insulin resistance decreases the catabolism of chylomicron remnants, leading to higher plasma apoB-48 concentrations [18]. Insulin resistance likely led to increased chyle in our patient by increasing her chylomicron production as well as impairing her ability to catabolize chylomicrons.

The increased chyle production due to insulin resistance was likely not the only contributing factor to her persistent chylothorax. Insulin resistance has a pathologic effect on the lymphatic vessels as well, and that likely contributed to her chylothorax. Insulin resistance has been shown to cause dysfunction in the lymphatic endothelial cells, which includes endothelial barrier dysfunction and increased lymphatic vascular permeability [19]. Insulin resistance leads to increased lymphatic permeability, which leads to increased extravasation of lymphatic fluid [20], and, in our patient’s case with a thoracic duct insult, this led to the worsening of her chylothorax.

Insulin resistance has a multifactorial role in the pathogenesis of our patient's chylothorax. While her iatrogenic thoracic duct injury made her more susceptible to a chylous effusion, her metabolic dysfunction resulted in a pathologic state of increased chylous flow and lymphatic endothelial barrier dysfunction. As her insulin resistance improved, as shown by a decrease in her HOMA-IR, TyG index, and TG/HDL ratio (Table 1), we hypothesized that she was able to decrease chylomicron production and increase catabolism, which led to a decrease in chylous flow. In addition, increasing her insulin sensitivity improved lymphatic endothelial function, which further decreased lymphatic extravasation, leading to a resolution in her symptoms and radiographic evidence of disease.

## Conclusions

It is important to consider the role insulin resistance may play in the pathogenesis of chylothorax due to its effect on increased chylomicron production and increased extravasation of lymphatic fluid. A properly implemented ketogenic diet results in distinct metabolic changes, particularly dramatic triglyceride reduction and, most importantly, improvements in insulin resistance, leading to changes in chylomicron metabolism and lymphatic endothelial function. As such, a low-carbohydrate ketogenic diet may be the optimal conservative treatment for chylothorax and should be evaluated in additional patients.
